# Phased Treatment Strategies for Cerebral Ischemia Based on Glutamate Receptors

**DOI:** 10.3389/fncel.2019.00168

**Published:** 2019-04-26

**Authors:** Yongjun Sun, Xue Feng, Yue Ding, Mengting Li, Jun Yao, Long Wang, Zibin Gao

**Affiliations:** ^1^Department of Pharmacy, Hebei University of Science and Technology, Shijiazhuang, China; ^2^Hebei Research Center of Pharmaceutical and Chemical Engineering, Hebei University of Science and Technology, Shijiazhuang, China; ^3^Hebei University of Science and Technology, Shijiazhuang, China; ^4^Shijiazhuang Vocational College of Technology and Information, Shijiazhuang, China; ^5^Department of Family and Consumer Sciences, California State University, Long Beach, CA, United States; ^6^State Key Laboratory Breeding Base—Hebei Province Key Laboratory of Molecular Chemistry for Drug, Shijiazhuang, China

**Keywords:** cerebral ischemia, excitotoxicity, phased treatment strategies, glutamate receptors, glutamate receptor antagonist

## Abstract

Extracellular glutamate accumulation following cerebral ischemia leads to overactivation of glutamate receptors, thereby resulting in intracellular Ca^2+^ overload and excitotoxic neuronal injury. Multiple attempts have been made to counteract such effects by reducing glutamate receptor function, but none have been successful. In this minireview, we present the available evidence regarding the role of all types of ionotropic and metabotropic glutamate receptors in cerebral ischemia and propose phased treatment strategies based on glutamate receptors in both the acute and post-acute phases of cerebral ischemia, which may help realize the clinical application of glutamate receptor antagonists.

## Background

Following cerebral ischemia, rapid glutamate release combined with deficiency or reversal in glutamate uptake causes extracellular glutamate accumulation. Excessive glutamate overactivates glutamate receptors, leading to intracellular Ca^2+^ overload and excitotoxic neuronal injury. In consideration of the association between cell death and glutamate excitotoxicity in stroke, numerous attempts have been made to prevent neuronal damage by reducing glutamate receptor function. Unfortunately, attempts to use drugs of this type to treat stroke patients have failed, owing to either lack of efficacy or presence of side effects. In this review, the roles of all types of ionotropic and metabotropic glutamate receptors (iGluRs and mGluRs) in cerebral ischemia have been discussed (Table 1), followed by an elaboration of our views on glutamate receptor-based treatment strategies for cerebral ischemia. Finally, phased therapeutics strategies proposed here may significantly improve cerebral ischemia treatment.

**Table 1:** The roles of different glutamate receptors in cerebral ischemia.

Receptor types			Main effects	Typical references
iGluRs	NMDARs	GluN2A	Not determined	Liu et al. (2007), von Engelhardt et al. (2007) and Zhou et al. (2013)
		GluN2B	Pro-death	Liu et al. (2007), von Engelhardt et al. (2007) and Zhou et al. (2013)
		GluN2C	Not determined	Chung et al. (2016), Holmes et al. (2018) and Doyle et al. (2018)
		GluN2D	Pro-death	Bai et al. (2013) and Doyle et al. (2018)
		GluN3A	Pro-survival	Nakanishi et al. (2009), Lee et al. (2015) and Wang et al. (2013)
		GluN3B	No obvious effect	Wang et al. (2013)
	AMPARs	GluA2-containing AMPARs	Not reported	
		GluA2-lacking AMPARs	Pro-death	Gorter et al. (1997), Liu et al. (2004) and Liu et al. (2006)
	KARs	GluK1	Pro-survival	Xu et al. (2008) and Lv et al. (2012)
		GluK2	Pro-death	Pei et al. (2005) and Pei et al. (2006)
		GluK3	Not reported	
		GluK4	Pro-death	Lowry et al. (2013)
		GluK5	Not reported	
mGluRs	Group I	mGluR1	Pro-death	Xu et al. (2007)
		mGluR5	Not determined	Bao et al. (2001), Szydlowska et al. (2007), Takagi et al. (2012) and Li et al. (2013)
	Group II	mGluR2	Pro-death	Corti et al. (2007), Motolese et al. (2015) and Mastroiacovo et al. (2017)
		mGluR3	Pro-survival	Corti et al. (2007)
	Group III	mGluR4	Pro-survival	Moyanova et al. (2011)
		mGluR6	Not reported	
		mGluR7	Pro-survival	Domin et al. (2015)
		mGluR8	Not reported	

## Roles of iGluRs in Cerebral Ischemia

iGluRs consist of N-methyl-D-aspartate receptors (NMDARs), α-amino-3-hydroxy-5-methyl-4-isoxazole propionate receptors (AMPARs), and kainate receptors (KARs). The overactivation of iGluRs triggered by excessive glutamate release plays a key role in ischemia-induced neuronal damage by enhancing intracellular calcium levels (Amantea and Bagetta, 2017).

### NMDARs

NMDARs, a type of ligand-gated and Ca^2+^-permeable ion channel, are widely expressed in the brain and play a key role in numerous physiological and pathophysiological processes. They are formed by combining different subunits (GluN1, GluN2A–D, and GluN3A–B) into tetrameric complexes (Hansen et al., 2018). Available evidence suggests that different types of NMDARs play different roles in cerebral ischemia.

#### GluN1 Subunit

Because GluN1 is the obligate subunit of NMDARs, the function of GluN1 is in line with that of NMDARs and the direct inhibition of GluN1 has neuroprotective effects. Notable compounds related to GluN1 are glycine-binding site antagonists, such as gavestinel and licostinel, which showed strong neuroprotective effects against ischemic neuronal damage *in vivo* (Lai et al., 2014). Besides the glycine-binding site, intervention on other domains may have neuroprotective effects. Macrez reported that a polyclonal antibody against amino acid sequences 19–371, inhibiting the interaction of tissue-type plasminogen activator (tPA) with GluN1, prevented the proexcitotoxic effect of tPA and extended the therapeutic window of thrombolysis (Macrez et al., 2011).

#### GluN2A-Containing Receptors

Although the role of GluN2A-containing receptors in cerebral ischemia has been extensively studied, it remains a controversial issue. Some researchers believe that activation of GluN2A-containing receptors is beneficial. The evidence consistent with this view is that application of an antagonist of GluN2A-containing NMDARs, NVP-AAM077, could exacerbate NMDA- or DL-threo-betahydroxyaspartate-induced excitotoxicity (Liu et al., 2007; Choo et al., 2012; Zheng et al., 2012), enhance oxygen-glucose deprivation (OGD)-induced neuronal apoptosis (Liu et al., 2007), and increase ischemic damage after transient focal or global ischemia. However, others have a contrasting view. It has been reported that knockdown of GluN2A attenuated NMDA- or middle cerebral artery occlusion (MCAO)-induced neuronal damage (Morikawa et al., 1998; Zhou et al., 2013). Additionally, antagonizing GluN2A-containing receptors with NVP-AAM077 or Zn^2+^ reduced NMDA-induced excitotoxicity in older (≥21 days *in vitro*) cortical or hippocampal culture (von Engelhardt et al., 2007; Stanika et al., 2009; Zhou et al., 2013). Enhanced tyrosine phosphorylation of GluN2A may be involved in the excitotoxic process (Yan et al., 2012). Among all the tyrosine kinases, cyclin-dependent kinase 5 (Cdk5) plays a key role in GluN2A phosphorylation, and perturbing interactions between Cdk5 and GluN2A abolished GluN2A phosphorylation and protected CA1 pyramidal neurons from ischemic insult (Wang et al., 2003). In view of the GluN2A signaling-pathway characteristics, we consider that GluN2A may play different roles at different times in cerebral ischemia, that is inducing neuronal death in the acute stage and promoting neuronal survival thereafter (Sun et al., 2018).

#### GluN2B-Containing Receptors

Overactivation of GluN2B-containing receptors is an important contributor to ischemic neuronal death (Sun et al., 2015). There is ample evidence that selective GluN2B antagonists, such as ifenprodil, CP-101, 606, Ro 25–6981, and Co 101244, prevented NMDA-mediated toxicity (Liu et al., 2007; von Engelhardt et al., 2007; Stanika et al., 2009; Choo et al., 2012; Zhou et al., 2013). Moreover, molecular knockdown of GluN2B could attenuate NMDA-induced neuronal death in cultured cortical neurons (Liu et al., 2007; Zhou et al., 2013). It has also been reported that ifenprodil reduced 4-vessel occlusion-triggered ischemic cell death (Chen et al., 2008). The significant pro-death effect of GluN2B can be largely attributed to its distinctive C-terminal domains (Martel et al., 2012).

Phosphorylation of GluN2B after cerebral ischemia may enhance its function and aggravate ischemic brain injury (Sun et al., 2016). After recruited to GluN2B C-terminal, CaMKIIα phosphorylated GluN2B at Ser1303 and up-regulated GluN2B function (Ahmed et al., 2017). Tat-CN21 could significantly reduce ischemic brain damage *via* inhibiting CaMKII binding to GluN2B (Vest et al., 2010; Ahmed et al., 2017). Thus, selectively inhibiting the phosphorylation of GluN2B may be a potential strategy for ischemia treatment.

Excessive activation of GluN2B-containing receptors could result in the activation of calpain, subsequently lead to the truncation of GluN2A and GluN2B in the C-terminal, and finally uncoupling NMDARs with downstream signaling proteins (Gascón et al., 2008). Strong blockage of GluN2B under this condition, which affects the normal signal transduction of NMDARs, may be detrimental.

#### GluN2C-Containing Receptors

It is not clear whether the activation of GluN2C**-**containing receptors is harmful to ischemic neurons. An early study showed that focal cerebral infarctions in GluN2C-knockout mice were significantly less extensive than those in wildtype mice (Kadotani et al., 1998). A recent study found that although GluN2C-knockout mice displayed similar infarct volumes compared to the wildtype mice, they showed decreased cerebral edema and enhanced neurological recovery (Holmes et al., 2018). Doyle et al. (2018) found that ischemic conditions could trigger the activation of GluN2C/2D-containing NMDARs in the oligodendrocytes under myelin sheath following the release of axonal vesicular glutamate into the peri-axonal space, and this process contributes to myelin damage. These results indicated the neurotoxic effect of GluN2C in cerebral ischemia. However, Chen and Roche (2009) reported that overexpression of GluN2C protected cerebellar granule cells from NMDA-induced toxicity. They also found that GluN2C-knockout mice exhibited greater neuronal death in the CA1 area of the hippocampus and reduced spatial working memory compared to the wildtype mice (Chung et al., 2016).

#### GluN2D-Containing Receptors

GluN2D-knockout mice showed reduced neuronal damage in NMDA-induced retinal ganglion cell death (Bai et al., 2013). The underlying mechanism may be related to myelin damage (Doyle et al., 2018).

#### GluN3A-Containing Receptors

Several studies have reported the neuroprotective effect of GluN3A. GluN3A knockout could increase cerebrocortical neuronal damage following NMDA application *in vitro*, NMDA-induced retinal ganglion cell death *in vivo*, or cortex damage in neonatal hypoxia-ischemia (Nakanishi et al., 2009). Subsequent experiments indicated that GluN3A knockout significantly increased the infarction volume in adult mice with ischemic stroke and hindered the sensorimotor functional recovery after stroke (Lee et al., 2015). It was also reported that overexpression of the GluN3A subunit in rat hippocampal neurons protected against OGD-induced toxicity (Wang et al., 2013).

#### GluN3B-Containing Receptors

The expression of GluN3B showed no visible changes following brain ischemia *in vivo* and OGD *in vitro* (Wang et al., 2013). Therefore, GluN3B might not be involved in the ischemic processes.

#### Expression of NMDAR Subunits Following Cerebral Ischemia

Cerebral ischemia could induce significant decreases in hippocampal GluN2A and GluN2B as early as 30 min, which may continue for several days (Zhang et al., 1997; Hsu et al., 1998; Dos-Anjos et al., 2009a,b; Liu et al., 2010; Fernandes et al., 2014; Han et al., 2016). While, the expression of GluN2C and GluN3A in the hippocampus was significantly increased following ischemia (Fernandes et al., 2014; Chung et al., 2016). Because the GluN2B/GluN2A ratio increases after ischemia, which may be detrimental to cell survival, upregulation of GluN2A expression may be helpful to ischemia treatment (Dos-Anjos et al., 2009b; Han et al., 2016).

#### NMDARs in Astrocytes

The NMDAR subunits expressed in astrocytes include GluN1, GluN2A, GluN2B, GluN2C, and GluN3A (Dzamba et al., 2015). However, the role of the NMDAR in astrocytes remains unclear. Alsaad et al. (2019) indicated that GluN2C may have a specific role in regulating glutamate release from astrocytes in response to glutamate spillover. Thus, the study of the roles of NMDARs on L-glutamate release in astrocytes may help to develop new therapeutic strategies.

#### Metabotropic NMDAR Signaling

Metabotropic NMDAR signaling, which is dependent on the allosteric movement of the C-terminal domain of NMDAR subunits (Dore et al., 2015), could mediate neuronal damage in the early stage of ischemia and disruption of this signaling *in vitro* or *in vivo* by administration of an interfering peptide was neuroprotective (Weilinger et al., 2016). Besides pro-death signaling, some metabotropic signaling mediated by NMDARs may be beneficial (Hu et al., 2016; Chen et al., 2017).

### AMPARs

Most fast excitatory transmission in the brain is mediated by AMPARs. They are composed of four different subunits (GluA1–4), and are homo- or hetero-tetrameric ion channels (Diering and Huganir, 2018). Due to the poor permeability of the GluA2 subunit to Ca^2+^, AMPAR Ca^2+^ conductance is dependent on the presence of GluA2. GluA2-containing AMPARs have low Ca^2+^ conductance, whereas GluA2-lacking AMPARs are Ca^2+^ permeable (Kwak and Weiss, 2006), and the latter are considered harmful. Detrimentally, cerebral ischemia could result in increased surface expression of GluA2-lacking AMPARs (Gorter et al., 1997; Liu et al., 2004, 2006). Many experiments have proven the neuroprotective effect of AMPAR antagonists such as perampanel (Nakajima et al., 2018), PNQX (Montero et al., 2007), EGIS-8332 (Matucz et al., 2006), GYKI 53405 (Matucz et al., 2006), and ZK 187638 (Elger et al., 2006) on ischemic injury. It should be emphasized that preventing the upregulation of GluA2-lacking AMPARs is an alternative treatment strategy. An interfering peptide with this effect could protect against neuronal damage induced by OGD (Wang et al., 2012) or transient MCAO (Zhai et al., 2013).

### KARs

Similar to NMDARs and AMPARs, KARs are tetramers assembled from a number of subunits (GluK1–5; Lerma and Marques, 2013). The GluK1–GluK3 subunits or GluK1–GluK3 combined with GluK4 or GluK5 subunits form functional homomeric or heteromeric receptors (Crepél and Mulle, 2015). Among all the KAR subunits, GluK2 might be a major contributor to ischemia-induced neuronal death. It was originally reported that administration of GluK2 antisense oligodeoxynucleotides once per day for 3 days before cerebral ischemia significantly decreased neuronal degeneration (Pei et al., 2005). Tat-GluK2-9c, a fusion peptide containing the Tat peptide and C-terminus peptide of GluK2, could interfere with the interaction of GluK2 with PSD-95 and suppress the formation of the GluK2-PSD-95-MLK3 triplicate complex, thereby preventing brain injury caused by cerebral ischemia (Pei et al., 2006). Besides GluK2, GluK4 also promotes the neuronal damage process. GluK4-knockout mice showed significant neuroprotection in the CA3 region of the hippocampus following intrahippocampal injection of kainate and widespread neuroprotection throughout the hippocampus following hypoxia-ischemia (Lowry et al., 2013). Contrary to the roles of GluK2 and GluK4, GluK1 may be a neuroprotective factor. ATPA, a selective GluK1 agonist, had a neuroprotective effect against ischemia/reperfusion-induced neuronal cell death, while the selective GluK1 antagonist, NS3763, or GluK1 antisense oligodeoxynucleotides had opposite effects (Xu et al., 2008; Lv et al., 2012).

## Roles of mGluRs in Cerebral Ischemia

mGluRs consist of eight receptor subunits (mGluR1–8), which are divided into three groups according to structural homology, pharmacologic profile, and signaling transduction pathways, namely group I (mGluR1 and mGluR5), group II (mGluR2 and mGluR3), and group III (mGluR4 and mGluR6–8). Compared with iGluRs, mGluRs play a more complicated role in cerebral ischemia.

### Group I mGluRs

#### mGluR1

Following cerebral ischemia, the activation of mGluR1 receptors may be harmful to neurons. Although knockout of the mGluR1 gene in mice could not limit the extent of ischemic brain injury (Ferraguti et al., 1997), a great number of studies have shown the neuroprotective effects of selective mGluR1 antagonists, such as LY367385 (Bruno et al., 1999; Murotomi et al., 2008; Li et al., 2013), YM-202074 (Kohara et al., 2008), EMQMCM (Szydlowska et al., 2007), CBPG (Pellegrini-Giampietro et al., 1999a,b; Cozzi et al., 2002; Meli et al., 2002), 3-MATIDA (Cozzi et al., 2002; Moroni et al., 2002), BAY 36-7620 (De Vry et al., 2001), and AIDA (Pellegrini-Giampietro et al., 1999a,b; Meli et al., 2002), on ischemic damage *in vivo*. One typical pro-death mechanism may be calpain-mediated mGluR1a truncation in the C-terminal domain (Xu et al., 2007). The truncated mGluR1a, which loses the ability to activate the neuroprotective PI3K-Akt signaling pathways and maintains the ability to increase the cytosolic calcium level, is an important contributor to excitotoxicity (Xu et al., 2007). The Tat-mGluR1 peptide consisting of the transduction domain of the Tat protein and the mGluR1a sequence spanning the calpain-cleavage site could protect mGluR1a from truncation and has exhibited neuroprotection against excitotoxicity both *in vitro* and *in vivo* (Xu et al., 2007; Zhou et al., 2009).

#### mGluR5

Unlike mGluR1, the role of mGluR5 in ischemia remains to be determined. Several articles have reported the neuroprotective effects of selective mGluR5 receptor antagonists, MPEP or MTEP, in transient global (Takagi et al., 2010, 2012) or focal cerebral ischemia (Bao et al., 2001; Szydlowska et al., 2007; Li et al., 2013). Other evidence indicated that CHPG, a selective mGluR5 receptor agonist, could protect cortical neurons and BV2 cells against *in vitro* traumatic injury (Chen et al., 2012) and OGD-induced cytotoxicity (Ye et al., 2017), respectively; it has shown protective effects against traumatic brain injury (Chen et al., 2012) or focal cerebral ischemia damage (Bao et al., 2001) *in vivo*. Moreover, mGluR5 has been shown to protect astrocytes from ischemic damage in the postnatal central nervous system (CNS) white matter (Vanzulli and Butt, 2015). However, other studies have found that antisense oligodeoxynucleotide directed to mGluR5, MPEP or MTEP, showed no neuroprotective effects against post-traumatic neuronal injury (Mukhin et al., 1996) *in vitro*, OGD damage (Meli et al., 2002), or hypoxia-ischemia injury in neonatal rats (Makarewicz et al., 2015). Moreover, the use of CHPG did not improve the functional and histological outcomes in a rat model of endothelin-1-induced focal ischemia (Riek-Burchardt et al., 2007).

### Group II mGluRs

mGluR2 and mGluR3 may mediate pro-death and pro-survival effects, respectively, in cerebral ischemia. Important evidence to this end was provided by a study showing that the neuroprotective effect of LY379268, a group II mGluR agonist, against NMDA-induced neuronal damage was lost in mice lacking mGluR3 receptors, while mGluR2 knockout enhanced the neuroprotective activity of LY379268 (Corti et al., 2007). Other experiments have shown that post-ischemic oral treatment with ADX92639, a selective negative allosteric modulator of the mGluR2 receptor, was highly protective against a 4-vessel occlusion model of transient global ischemia in rats, while the administration of the mGluR2 receptor agonist, LY487379, aggravated ischemia-induced neuronal damage in both the CA1 and CA3 regions (Motolese et al., 2015). Moreover, genetic deletion of mGluR2 receptors could improve the short-term outcome of cerebral transient focal ischemia (Mastroiacovo et al., 2017).

### Group III mGluRs

The activation of group III mGluRs may be beneficial to ischemic neurons. Although early research indicated that (R, S)-4-phosphonophenylglycine, a selective group III mGluR agonist, had no significant influence on neuronal damage in both focal and global cerebral ischemia models (Henrich-Noack et al., 2000), group III mGluR agonist, ACPT-I, showed neuroprotective effects against MCAO/reperfusion in both normotensive (Domin et al., 2014) and spontaneously hypertensive (Domin et al., 2018) rats. It was also reported that the absence of mGluR4 receptors and PHCCC, a positive allosteric modulator of mGluR4 receptors, could enhance and reduce brain damage, respectively, induced by permanent MCAO or endothelin-1-induced transient focal ischemia (Moyanova et al., 2011). Additionally, AMN082, an allosteric agonist of mGluR7, protected neurons against OGD- or kainate-induced damage (Domin et al., 2015).

### Phased Treatment Strategies for Cerebral Ischemia Based on Glutamate Receptors

**Figure 1:**
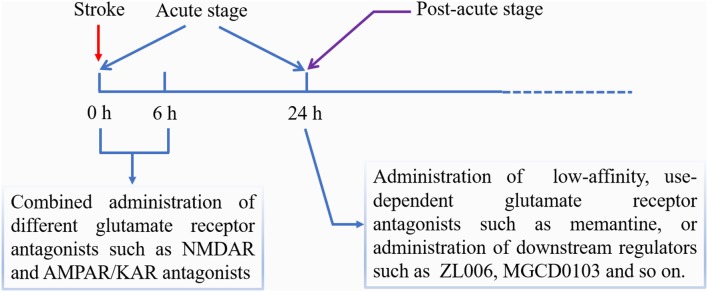
Phased treatment strategies for cerebral ischemia based on glutamate receptors.

The diagram of phased treatment strategies for cerebral ischemia based on glutamate receptors is shown in Figure 1.

### Treatment Strategies in the Acute Phase

Because several types of glutamate receptors contribute to ischemic damage in the acute phase, we can adopt a “hitting hard” strategy through the combined use of different antagonists in order to improve the curative effect. Several articles have reported that combined use of NMDAR and AMPAR antagonists, such as MK-801 and NBQX (or CNQX), could achieve additive protective effect in both *in vitro* and *in vivo* ischemia models (Mosinger et al., 1991; Lippert et al., 1994; Virgili et al., 1995). However, this combination strategy may have a greater impact on physiological function, making it difficult to use in the clinic (McManigle et al., 1994). Therefore, we consider that new antagonists having the characteristics of short-acting and ischemic tissue-targeting should be developed in order to minimize the adverse reactions. In addition, combined use of iGluR and mGluR antagonist such as MK-801 and AP-3, showed a synergistic effect in the reduction of cell death induced by OGD (Zagrean et al., 2014).

### Treatment Strategies in the Post-acute Phase

In the post-acute phase, the major role of glutamate receptors may be promoting neuron survival. Under this condition, we can adopt a “precision treatment” strategy by selectively blocking the pro-death signaling or enhancing the pro-survival signaling. One method is to use low-affinity, use-dependent glutamate receptor antagonists, which do not interfere with the receptors’ physiological function. Consistent with this view is that delayed treatment with MK-801 did not show beneficial effects (Zhou et al., 2015), while, post-acute delivery of memantine promoted post-ischemic neurological recovery, peri-infarct tissue remodeling, and contralesional brain plasticity (Wang et al., 2017). Another method is to administer selective inhibitors of downstream pro-death signaling molecules. For instance, administration of histone deacetylase 2 inhibitors starting 5–7 days after stroke promoted recovery of motor function (Lin et al., 2017). Moreover, animals treated with Tat-HA-NR2B9c or ZL006 starting at 4 days after ischemia showed improved functional recovery (Zhou et al., 2015). In addition, considering the pro-survival effects of GluN2A-containing receptors and Group III mGluRs after cerebral ischemia, the agonists of these kinds of receptors may become alternative therapeutic drugs in the post-acute phase.

## Conclusions

Low efficacy or serious side effects limit the clinical application of glutamate receptor antagonists, which indicates the need to modify the existing treatment strategies. The new strategies proposed in this article may help realize the clinical application of glutamate receptor antagonists.

## Author Contributions

ZG and LW proposed the topic of the article, participated in literature search and revised the manuscript. YS participated in literature search and manuscript writing. XF, YD, ML and JY participated in manuscript writing. All authors read and approved the final manuscript.

## Conflict of Interest Statement

The authors declare that the research was conducted in the absence of any commercial or financial relationships that could be construed as a potential conflict of interest.
